# *Ab initio* study of defect interactions between the negatively charged nitrogen vacancy centre and the carbon self-interstitial in diamond

**DOI:** 10.1098/rsta.2023.0174

**Published:** 2024-01-22

**Authors:** Andrew R. Kirkpatrick, Guangzhao Chen, Helen Witkowska, James Brixey, Ben L. Green, Martin J. Booth, Patrick S. Salter, Jason M. Smith

**Affiliations:** ^1^ Department of Materials, University of Oxford, Oxford OX1 3PJ, UK; ^2^ Department of Engineering Sciences, University of Oxford, Oxford OX1 3PJ, UK; ^3^ Department of Physics, University of Warwick, Coventry CV4 7AL, UK; ^4^ Department of Engineering, University of Warwick, Coventry CV4 7AL, UK; ^5^ Department of Electrical and Computer Engineering, Michigan State University, East Lansing, MI 48824-126, USA

**Keywords:** diamond, quantum, defects, colour centres, *ab initio*

## Abstract

Fabrication techniques for nitrogen-vacancy centres in diamond require the creation of Frenkel defects (vacancy-interstitial pairs) the components of which can interact with formed NV centres affecting their photophysical properties. Here we use Density Functional Theory simulations of inter-defect electronic and strain interactions to explore how the NV centre and carbon self-interstitial interact in different configurations. We find that hybridization occurs between the NV centre e-orbitals and the carbon self-interstitial when an interstitial is present on the vacancy side of the NV centre. We propose that this phenomenon may explain the fluorescence blinking of NV centres observed during annealing.

This article is part of the Theo Murphy meeting issue ‘Diamond for quantum applications’.

## 1. Introduction

**Figure 1.**
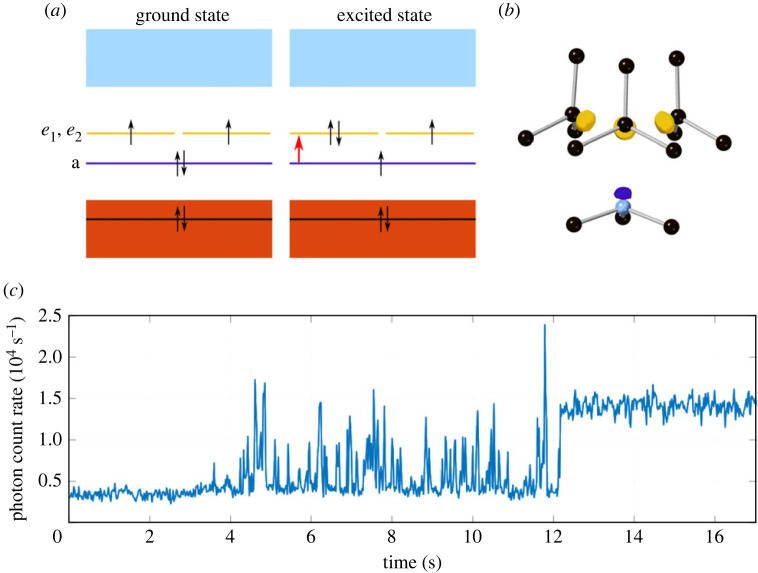
(*a*) The band structure of the NV centre in diamond, the red arrow depicts the $a_{1}^{2}a_{2}^{2}e_{1}^{1}e_{2}^{1}\to a_{1}^{2}a_{2}^{1}e_{1}^{2}e_{2}^{1}$ transition which results in fluorescence. (*b*) A diagram of the NV centre with the orbitals colour coded with (*a*). (*c*) An example of the fluorescence intensity trace during NV the laser annealing process demonstrating the intermittent fluorescence spiking.

The negatively charged nitrogen-vacancy (NV) defect centre in diamond has in recent years become an important component for quantum sensing [1] and shows considerable promise for advanced applications in quantum communications and computing [2]. The centre’s spin-dependant optical transitions (figure 1*a*) and inter-system crossing between triplet and singlet spin manifolds provide a convenient means of optical initialization and readout of the spin state [3]. The electron spin coherence time of the NV${\;}^{-}$ is typically of order milliseconds at room temperature [4–6].

The generation of NV centres in diamond involves the creation of vacancies via ion implantation [7], e-beam irradiation [8] or laser processing [5]. The diamond is then annealed such that the vacancies migrate to form an NV centre with a substitutional nitrogen atom, which has either been incorporated during the diamond growth or implanted prior to annealing. The annealing step is also critical in removing damage from the diamond lattice which would otherwise compromise the properties of the NV centres, and significant effort has gone into understanding how different types of lattice damage respond to thermal annealing [9,10]. The effects of different types of defect on NV centre properties have also been studied [11].

The generation of single NV centres at room temperature in an optical microscope using femtosecond laser pulses has allowed the observation of dynamics in the fluorescence during NV formation, the origins of which are not yet well understood [12]. In that work, a single laser pulse was used to generate Frenkel defects (vacancy-interstitial pairs) followed by a train of lower energy pulses to excite the diffusion of vacancies. The signature of the formation of an NV centre was an initial period of intermittent fluorescence followed by a sudden stabilization (figure 1*c*). This behaviour suggests that when the NV centre is initially formed the presence of other defects in the vicinity modifies its fluorescence, but that these additional defects eventually either diffuse away or somehow become inactive rendering stable NV emission.

Here we present a theoretical study of interactions between an NV centre and a carbon self-interstitial, performed using Density Functional Theory (DFT). The purpose of the study is to simulate the situation in which an NV centre is formed from a substitutional nitrogen and a single Frenkel defect, leaving a carbon self-interstitial nearby. This situation is expected to occur in laser processing when the laser pulse energy is tuned to create minimal lattice damage—the dynamics of Frenkel defect generation using laser processing were reported previously [13]. The remaining carbon self-interstitial is assumed to be oriented along an ⟨001⟩ axis of the crystal which is the only stable self-interstitial configuration in diamond [14,15]. The interstitial consists of two carbon atoms bonded along the ⟨001⟩ axis centred on a lattice site, with each carbon atom bonded to an additional two carbons and its fourth valence electron occupying a p-orbital. The ⟨001⟩ carbon self-interstitials are relatively mobile and diffuse through the diamond lattice with an activation energy of 1.65 eV [16].

We consider the influence on the NV centre both of strain due to the carbon self-interstitial and of orbital hybridization between the NV centre e-orbitals and the carbon self-interstitial p-orbitals. The conclusion drawn is that these phenomena could explain the observed fluorescence dynamics during NV formation.

## 2. Methods

Density Functional Theory (DFT) calculations were performed using the Perdew–Burke–Ernzerhof (PBE) functional [17] in CASTEP [18]. Simulations were of a 3×3×3 (216 atoms) and a 3×3×6 (432 atoms) supercell of the diamond cubic unit cell. This was to ensure the super cells were sufficiently large enough that the results were free of the effects of interactions between supercells via the periodic boundary conditions. The formation energy of the NV centre was converged to provide an appropriate value for the Monkhorst–Pack (MP) grid spacing [19] and the plane wave basis size. The converged formation energy, $E_{f}$ was calculated using equation (2.1):

2.1 $$E_{f}=E_{\mathrm{tot}}-(\mu_{N}+n_{C}\mu_{C}),$$

where $E_{\mathrm{tot}}$ is the total energy of the NV-containing supercell, $\mu_{N/C}$ is the chemical potential of nitrogen and carbon respectively, and $n_{C}$ is the number of carbon atoms in the supercell. The formation energy of the NV${\;}^{0}$ defect was calculated to be 6.83 eV, which is within 10% error of the formation energy calculated by HSE06 [20]. This error is expected since HSE06 is free of self-interaction errors in diamond, and is acceptably small for the purpose of this study. HSE06 itself was not considered for use within this article due to the scale of the study proposed and the functional’s comparatively large computational expense.

The MP grid was converged to a 3×3×3 mesh whereby decreasing the MP grid spacing to a 5×5×5 mesh led to a change in formation energy of <1 meV. The plane wave basis size was converged to 600 eV whereby increasing the basis to 650 eV led to a change in formation energy of greater than 7 meV. The diamond lattice parameter was calculated to be 3.59 Å.

## 3. Results

### (a) Strain interactions

In order to investigate strain field interactions the strain fields surrounding the carbon self-interstitial and NV centre were simulated. Each defect state was placed in an isolated 3×3×3 diamond supercell and the geometric ground state was calculated using Broyden–Fletcher–Goldfarb–Shanno (BFGS) geometry optimization. A geometry was considered optimized based on the minimization of four parameters, the two most relevant of which were the maximum change in any atomic position between steps and the maximum force on any atom. The tolerance to these parameters was set to 0.001 Å and 0.05 eV/Å, respectively. In the case of the carbon self-interstitial, the supercell used was expanded, along the defect axis, to 3×3×6 due to the range of its strain field. The effects of the strain field on the band structure of the NV${\;}^{-}$ centre were calculated by placing the NV${\;}^{-}$ centre in a 3×3×3 supercell and applying the appropriate lattice deformation to the supercell.

**Figure 2.**
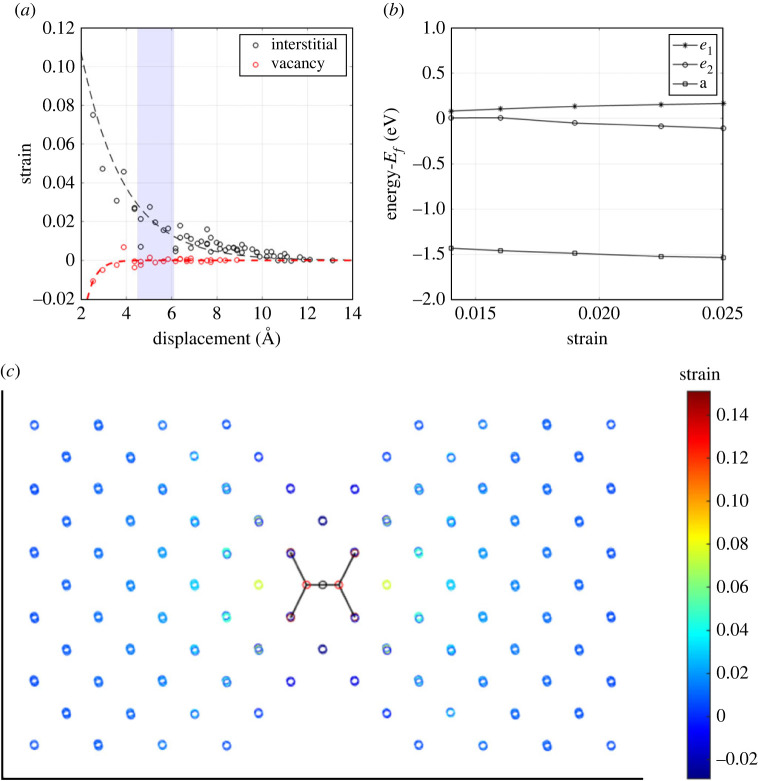
(*a*) The simulated strain field in the diamond lattice surrounding a NV and ⟨100⟩-split carbon self-interstitial. The interstitial strain field is samples along the defect axis due to its high directionality. (*b*) The behaviour of the NV centre’s band gap energy levels as a function of lattice strain. The strain sampled corresponds to a spatial separation, between the NV and ⟨100⟩-split carbon self-interstitial, shown by the shaded region in figure (*a*). (*c*) A diagram of the compressive strain field surrounding the ⟨100⟩-split carbon self-interstitial.

Figure 2*a* shows how the magnitude of the strain field parallel to the axis of the NV centre and the magnitude of the strain field along the axis of carbon self-interstitial vary with distance from the respective defects. The NV centre demonstrates a small localized strain field close to the defect, while the interstitial strain field is less localized and of larger magnitude. Figure 2*c* shows the two-dimensional distribution of this field. These results suggest that the NV centre will experience lattice strain of order 2% or more when a carbon self-interstitial is within 10 Å, and that the strain it experiences will depend strongly on the orientation of the interstitial.

The effect of lattice strain on the electronic structure of NV${\;}^{-}$ has been well documented both theoretically [21,22] and experimentally [23,24]. Broadly, the strain Hamiltonian separates into terms which preserve the defect’s threefold rotational symmetry and those which break it: the former lead to shifts in the optical transitions while the latter lift the degeneracy of the $e_{x,y}$ orbitals and cause a splitting in the transition energy. This latter effect is shown in figure 2*b*. The range of strains plotted corresponds to an NV-$C_{i}$ separation between 5 Å and 8 Å as indicated with the shaded region in figure 2*a*. A splitting is observed between the $e_{x}$ and $e_{y}$ states, but otherwise the electronic structure remains unchanged. When the carbon self-interstitial is between 4.5 Å of the NV centre the energy levels are split by 75 meV, however, the splitting rapidly increases, as the defect separation approaches 6.1 Å to 280 meV.

### (b) Electronic interactions

Electronic interactions between configurations of the NV centre and carbon self-interstitial were simulated by generating all symmetrically unique second and third nearest neighbour configurations of the two defects in the diamond supercell. After geometry optimization of each complex their new structures were used to calculate band structures. The c2x programme was then used to extract volumetric orbital data from the band structures [25].

**Figure 3.**
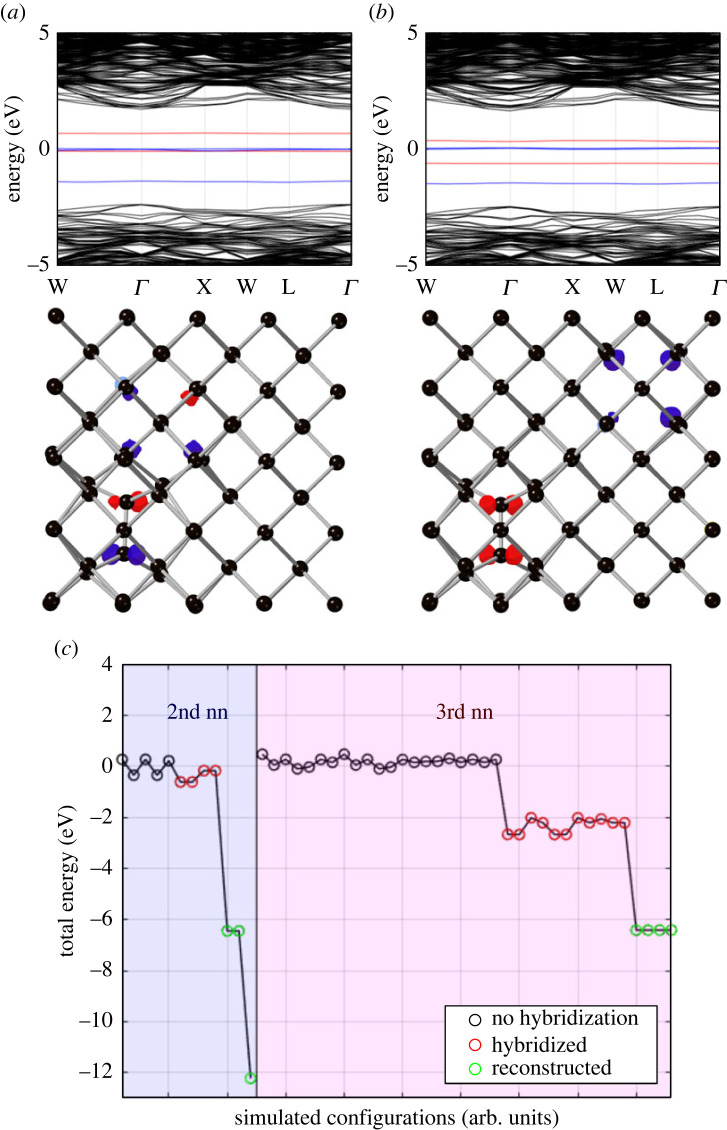
Band structure and orbital population plots of the electronic interactions between a NV${\;}^{-}$ centre and ⟨100⟩-split carbon self-interstitial in diamond, where (*a*) the defect centres have hybridized and (*b*) where they have not. Hybridization occurs between the NV centre’s e-manifold and the π-orbitals of the interstitial. In both plots, the energy levels in the band structure and the orbital populations have been colour coded blue for the NV centre and red for the interstitial. (*c*) A plot of the energy difference between each simulated configuration of the two defect centres, where reconstruction and hybridization occur with a distinct drop in the energy compared to unhybridized configurations.

By examining the isolated band structures of the NV centre and the $C_{i}$ we can identify that electronic interactions are likely to occur between the energetically similar NV e-manifold and the degenerate π-orbitals of the $C_{i}$. In particular, certain configurations of the carbon self-interstitial and the NV centre demonstrate hybridization between the NV${\;}^{-}$ e-orbitals and the $C_{i}$ π-orbitals. This can be seen in figure 3*a*,*b* for hybridized and non-hybridized cases, respectively. Out of the 48 configurations simulated, 8 lead to reconstruction and 15 result in hybridization. The π-orbital involved in hybridization is usually the closer of the two to the NV centre. Hybridization does not occur with the NV${\;}^{-}$ a-orbital since it is energetically distant from the carbon self-interstitial π-orbitals. It is also noted that nearly every configuration results in splitting of the interstitials’ degenerate energy states, which is of greater magnitude the closer the two defects are to each other.

Figure 3*c* shows the relative energies of the non-hybridized, hybridized and reconstructed configurations. For second nearest-neighbours hybridization lowers the energy only very slightly, while reconstruction lowers the energy by over 6 eV. For third nearest-neighbours, interestingly, hybridization lowers the energy by some 2 eV and reconstruction, again, by 6 eV.

**Figure 4.**
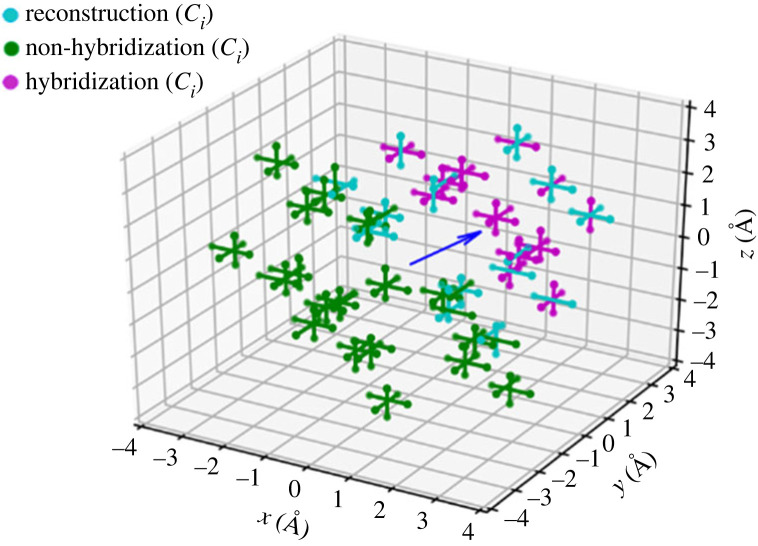
Diagram of ⟨100⟩-split carbon self-interstitial positions around the NV centre. Each of these interstitials are colour coded to demonstrate if their presence results in hybridization or reconstruction with the NV centre. The nitrogen atom and vacancy of the NV centre is marked in blue and red, respectively.

Figure 4 shows a map of all of the positions configurations of the carbon self-interstitial that results in hybridization with the NV. It can be seen from this figure that all configurations that result in either hybridization or reconstruction of the diamond lattice are configurations in which the interstitial is on the vacancy side of the NV centre. Geometrically this is consistent with the localization of these orbitals which are formed by anti-bonding between the dangling bonds that point into the vacancy from the three carbon atoms that surround it.

## 4. Discussion

The strain interactions between the carbon self-interstitial and NV centre would be expected to perturb NV fluorescence slightly but not to cause any substantial quenching. By contrast, we propose that electronic hybridization between the NV centre e-orbitals and the carbon self-interstitial π-orbitals would lead to the creation of strong non-radiative relaxation pathways via the interstitial, which would quench the NV fluorescence.

The dependence of orbital hybridization on the position of the interstitial relative to the NV centre suggests that fluorescence intermittency could result from changes in quenching as the interstitial diffuses through the lattice. Figure 4 reveals that for some interstitial locations, and in particular for sites close to the equatorial plane of the NV centre, a single hop of the interstitial to an adjacent site can switch between hybridized and non-hybridized states and would be expected to yield a sudden change in fluorescence intensity. The high mobility of the interstitial (the energy barrier to hopping for which is 1.68 eV) would be expected to result in significant intermittency before the interstitial diffuses far enough away that the NV emission becomes stable.

A further possible explanation for fluorescence intermittency stems from the finding that hybridization only occurs when the interstitial is on the vacancy side of the NV centre. Inversion of the NV centre by the substitutional nitrogen migrating into the vacancy’s position occurs with a barrier height of 4.8 eV [11,26,27], less than the energy barrier height for NV${\;}^{-}$ formation and also less than the band gap of diamond.

Finally we note that the relative energies of the hybridized and reconstructed states shown in figure 3*a* suggest that diffusion with modest energy (5 eV) allows full movement between these states, although it might be that a reconstructed state must evolve into a hybridized state before it becomes a non-hybridized state. Figure 4 suggests that all reconstructed states are in close proximity to hybridized states and so it does not appear that the interstitial would become trapped in a reconstructed state with no means of escape.

If correct, these proposed mechanisms have significant consequences for NV fabrication using laser processing. The high mobility of the interstitial allows for straightforward diffusion of the defect far away from the NV${\;}^{-}$ to leave a defect-free, low strain environment in which the NV${\;}^{-}$ properties are optimized.

## 5. Conclusion

In this article, both strain and electronic interactions between NV${\;}^{-}$ and the carbon self-interstitial in diamond have been examined using DFT in order to investigate the origin of the transient fluorescent signal found during the laser fabrication of NV centres. Strain interactions have demonstrated to be dominated by the interstitial carbon and do not appear to perturb the NV band structure enough to disrupt fluorescence. Investigating the electronic structure of configurations of the ⟨100⟩-split carbon self-interstitial and NV centre demonstrates that hybridization can occur between the NV’s e-manifold and the energetically similar π-bonds of the interstitial. This is of particular relevance when considering the NV centres fluorescence since the 637 nm fluorescent transition occurs from this e-manifold. It is proposed that such hybridization may cause non-radiative channels for the excited electron to return to the ground state, effectively terminating the fluorescence of the NV centre.

## Data Availability

For review, data are available here: https://drive.google.com/file/d/1g9VtjieziMMOWXRzZXUoQh1Xghy02ddJ/view?usp=sharing and http://dx.doi.org/10.5281/zenodo.22558 [28]. Data available from the Dryad Digital Repository: [29].
